# Neurons are the Primary Target Cell for the Brain-Tropic Intracellular Parasite *Toxoplasma gondii*

**DOI:** 10.1371/journal.ppat.1005447

**Published:** 2016-02-19

**Authors:** Carla M. Cabral, Shraddha Tuladhar, Hans K. Dietrich, Elizabeth Nguyen, Wes R. MacDonald, Tapasya Trivedi, Asha Devineni, Anita A. Koshy

**Affiliations:** 1 BIO5 Institute, University of Arizona, Tucson, Arizona, United States of America; 2 Department of Immunobiology, University of Arizona, Tucson, Arizona, United States of America; 3 Undergraduate Biology Research Program, University of Arizona, Tucson, Arizona, United States of America; 4 Department of Neurology, University of Arizona, Tucson, Arizona, United States of America; University of New Mexico, UNITED STATES

## Abstract

*Toxoplasma gondii*, a common brain-tropic parasite, is capable of infecting most nucleated cells, including astrocytes and neurons, *in vitro*. Yet, *in vivo*, *Toxoplasma* is primarily found in neurons. *In vitro* data showing that interferon-γ-stimulated astrocytes, but not neurons, clear intracellular parasites suggest that neurons alone are persistently infected *in vivo* because they lack the ability to clear intracellular parasites. Here we test this theory by using a novel *Toxoplasma*-mouse model capable of marking and tracking host cells that directly interact with parasites, even if the interaction is transient. Remarkably, we find that *Toxoplasma* shows a strong predilection for interacting with neurons throughout CNS infection. This predilection remains in the setting of IFN-γ depletion; infection with parasites resistant to the major mechanism by which murine astrocytes clear parasites; or when directly injecting parasites into the brain. These findings, in combination with prior work, strongly suggest that neurons are not incidentally infected, but rather they are *Toxoplasma*’s primary *in vivo* target.

## Author Summary

*Toxoplasma gondii* is a ubiquitous intracellular parasite that chronically infects the brain of up to 1/3 of the world’s population. While most infected persons have no symptoms, *Toxoplasma*’s tropism for the brain underlies the parasite’s ability to cause debilitating neurologic disease in the developing fetus and the immunocompromised, such as AIDS or organ transplant patients. Our understanding of which cells of the brain interact with and are infected by *Toxoplasma* primarily comes from work in tissue culture, which cannot re-capitulate the complexity of a living animal. Here, we overcome this barrier by studying brain-*Toxoplasma* interactions using a novel mouse model that allows us to permanently mark and track brain cells that have interacted with parasites. Contrary to what has been shown in cell culture, we find that throughout acute and chronic brain infection *Toxoplasma* almost exclusively interacts with neurons, and rarely interacts with astrocytes. This neuronal predilection persists even in highly manipulated circumstances such as the introduction of parasites directly into the brain. This work is the first to show that *Toxoplasma* primarily interacts with neurons *in vivo*, which suggests that to understand *Toxoplasma*’s ability to persistently infect the brain, we must understand the neuron-*Toxoplasma* interaction.

## Introduction

Host cell-microbe interactions govern the survival and propagation of intracellular microbes. The importance of these interactions is particularly pronounced in chronic infections where persistence in specific cell types eludes pharmacologic or immunologic cures, leaving the pathogen to reactivate at opportunistic times. Understanding host cell-pathogen dynamics–from how pathogens find permissive cells to how they manipulate those cells–will provide opportunities to develop therapies that eliminate currently incurable persistent pathogens.

Here, we seek to address how the ubiquitous intracellular parasite *Toxoplasma gondii* establishes a persistent brain infection. *Toxoplasma* is estimated to chronically infect the central nervous system (CNS) of up to 1/3 of the world’s population. This chronic CNS infection, for which there are no curative therapies, underlies *Toxoplasma*’s ability to reactivate and cause devastating neurologic disease and death in the immunosuppressed [1–3]. Prior to the introduction of effective combination anti-retroviral therapy, *Toxoplasma* was the most common cause of focal neurologic disease in AIDS patients [4]. Even today, toxoplasmic encephalitis occurs in untreated or undiagnosed AIDS patients as well as in patients on newer immunomodulants [3,5–7].

Our understanding of *Toxoplasma*-CNS host cell interactions comes primarily from *in vitro* studies and the mouse model of toxoplasmosis. Like humans, mice are natural intermediate hosts in which the CNS is the major organ of encystment [8,9]. Studies in human and rodent primary CNS cell cultures have established that *Toxoplasma* is capable of infecting and encysting in both astrocytes and neurons [10–12], two major parenchymal CNS cell types implicated in chronic infection. Yet, *in vivo*, *Toxoplasma* almost exclusively persists in neurons [13–15].

One of two alternative mechanisms could most easily reconcile these divergent *in vitro* and *in vivo* host cell preferences. One possibility is that both astrocytes and neurons are infected *in vivo* but only infected astrocytes either kill or are killed by the parasite, leaving neurons as the primary host cell for persistent infection. Alternatively, *Toxoplasma* could primarily interact with neurons *in vivo*, meaning that *Toxoplasma* persists in neurons because these are the cells with which the parasite predominantly interfaces. In support of the “astrocyte-killing” possibility, prior *in vitro* work established that stimulation with interferon-gamma (IFN-γ), the major cytokine required for systemic or cerebral resistance to *Toxoplasma* [16,17], causes astrocytes, but not neurons, to use IFN-γ-regulated GTPases to kill up to 90% of the intracellular parasites [18–21]. While these studies imply that *Toxoplasma* primarily encysts in neurons because neurons cannot clear intracellular parasites, no studies have directly addressed this question *in vivo*, in part because of a lack of techniques to identify host cells with transient parasite interactions (e.g. transiently infected astrocytes that killed the intracellular parasite).

We overcame this barrier by generating *Toxoplasma* strains that inject Cre recombinase into host cells [22]. Using these *Toxoplasma*-Cre strains to infect mice that express GFP in a Cre-dependent manner [23] enables the identification and tracking of host cells that directly interact with parasites. Importantly, this system causes a parasite-triggered permanent genetic change in host cells but does not require active infection. Therefore, host cells are permanently marked even in situations in which the host cell is injected with parasite proteins but is not invaded or in which the parasite invades and is subsequently killed by the host cell [24,25]. Our prior work using this system revealed that at three weeks post-infection, the majority of the marked parenchymal CNS cells (GFP^+^ cells) appeared to be uninfected neurons [24]. As this finding was contradictory to what would be predicted by the “astrocyte-killing” model of CNS-*Toxoplasma* interactions, the work here expands upon those initial findings to more clearly define which parenchymal CNS cells directly interact with parasites *in vivo*. Remarkably, during CNS infection with two genetically divergent *Toxoplasma*-Cre strains, we find that neurons consistently make up the majority of the GFP^+^ parenchymal CNS cells regardless of time point or infecting strain. This neuronal predilection remains even in highly manipulated circumstances such as IFN-γ depletion or when infecting with parasites resistant to a major mechanism by which murine astrocytes eliminate intracellular parasites *in vitro*. Finally, imaging whole infected neurons *in situ* reveals that cysts are predominantly found in the extensive network of neuronal processes, not the neuron cell bodies. Collectively, these data highly suggest that *Toxoplasma*’s persistence in neurons is driven by a strong *in vivo* predilection for interacting almost exclusively with neurons and that physical properties such as the extensive size and breadth of neuronal processes may play a role in determining this *in vivo* preference.

## Results

### Neurons are the primary CNS parenchymal cell injected with *Toxoplasma* effector proteins throughout CNS infection

**Fig 1:**
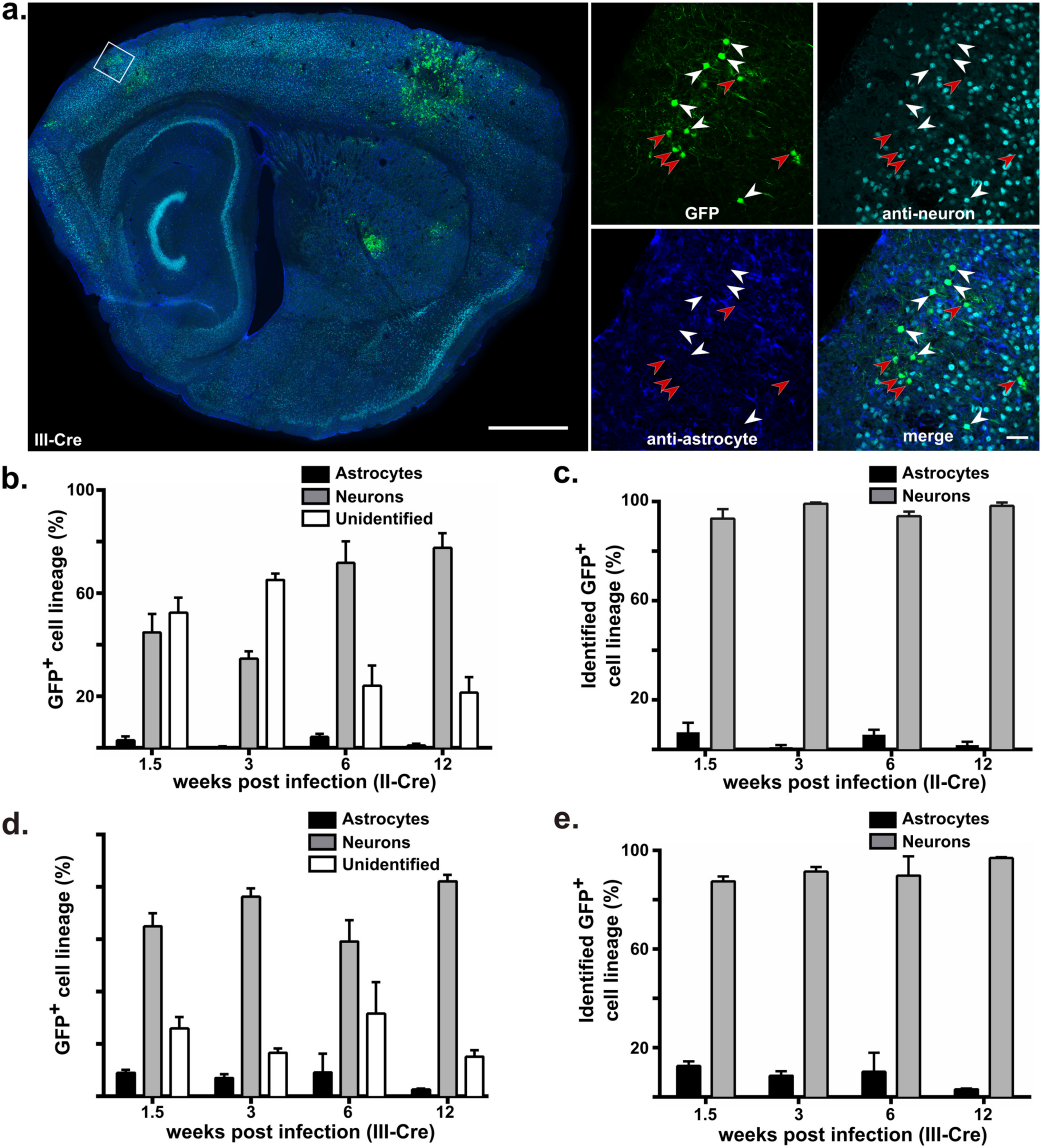
*Toxoplasma* parasites predominantly interact with neurons throughout CNS infection. Cre-reporter mice were infected with II-Cre or III-Cre *Toxoplasma* parasites as labeled. Brains were harvested, sectioned, and stained for neurons (anti-neuronal cocktail) and astrocytes (anti-GFAP) at specified time points. Stained sections were analyzed by confocal microscopy to identify if GFP co-localized with stains for neurons, astrocytes, or neither (unidentified). (a) Representative stitched-grid image of a brain section from a III-Cre infected mouse at 3 weeks post infection (wpi). White boxed area in left image is enlarged and separated into the different channels, as labeled (right images). White arrowheads denote GFP^+^ cells that co-localized with anti-neuron staining, red arrowheads denote GFP^+^ cells that did not co-localize with either anti-neuron or anti-astrocyte staining. Left image scale bar, 1 mm. Enlarged image scale bar, 50 μm. (b) Quantification of co-localization for II-Cre infected brain sections at different time points post infection. Bars, mean ±SEM. (c) As in (b) but restricting the analysis only to GFP^+^ cells identified as neurons or astrocytes. (d), (e) As in (b), (c) but for III-Cre infected mice. Bars, mean ±SEM. N = 3–4 mice/time point/*Toxoplasma* strain. The total number of GFP^+^ cells examined at each time point ranged from 232–372/II-Cre and 368–506/III-Cre. No statistical differences were found in the mean percentage of GFP^+^ neurons across time points in either II-Cre or III-Cre infection (one-way ANOVA, p = 0.13 and p = 0.45 respectively). At 6 wpi, one of the III-Cre infected mice had a substantially lower percentage of GFP^+^ neurons compared to the other mice (67 vs. 91,100,100). Exclusion of this mouse from data analysis changes the mean percentage of GFP^+^ neurons at 6 wpi from 90 ±8 (full data set) to 97 ±3 (1 mouse excluded), which results in a suggestion that in III-Cre infected mice the percentage of GFP^+^ neurons is lower at 1.5 wpi compared to 6 and 12 wpi (one-way ANOVA, p < 0.01). No GFP^+^ cells were found in II-Cre or III-Cre infected brain sections from 0.5 wpi (N = 2 mice/*Toxoplasma* strain, 9 sections/mouse).

To identify which CNS host cells directly interact with *Toxoplasma* and if those interactions are *Toxoplasma* strain-specific, we infected Cre reporter mice intraperitoneally with either of two genetically divergent strains of *Toxoplasma* (type II Prugniaud and type III CEP strains) engineered to express the fluorescent protein mCherry and to inject Cre into host cells [24,25]. For simplicity, these strains are referred to as II-Cre or III-Cre. Infected mice were sacrificed at 0.5, 1.5, 3, 6, and 12 weeks post-infection (wpi), and brains harvested, sectioned and stained with antibodies against neurons and astrocytes. Host cells were defined as cells that expressed GFP (and therefore had been injected with the parasite Cre fusion protein). To determine the lineage of the parenchymal GFP^+^ cells, we analyzed the stained brain sections with confocal microscopy and identified co-localization between GFP and neuronal or astrocytic staining (Fig 1a, S5 Fig provides labeled schematics of shown brain sections). We categorized GFP^+^ cells as neurons (Fig 1a white arrowhead, S1a Fig), astrocytes (S1b Fig), or unidentified, for any GFP^+^ cell that did not co-localize with neuronal or astrocytic stains (Fig 1a red arrowheads, S1a, S1c and S1d Fig). At 0.5 wpi, no GFP^+^ cells were found in the brain (N = 2 mice/*Toxoplasma* strain, 9 sections/mouse), which is consistent with prior reports of the timing of parasite entry into the CNS after peripheral infection [26,27]. Throughout the remaining time points (1.5–12 wpi) and with both II-Cre and III-Cre parasites, neurons made up the vast majority of the identified cells (Fig 1b–1e), representing over 85% of the identified GFP^+^ cells in both II-Cre (range 93–97%, Fig 1c) and III-Cre infections (range 87–97%, Fig 1e). In both infections, the highest percentage of GFP^+^ astrocytes (7 ±4% and 13 ±2%, II-Cre and III-Cre respectively) was seen at 1.5 wpi.

At 1.5 and 3 wpi, the unidentified cells made up a significant portion of the GFP^+^ cells, especially in the II-Cre infected mice (Fig 1b). Thus, we focused on ensuring that at these time points we were not missing significant numbers of GFP^+^ astrocytes or oligodendrocytes, the last of the major parenchymal CNS cell types. For astrocytes, we stained tissue both with our standard anti-GFAP antibody, the most commonly used marker for identifying astrocytes, as well as an additional antibody against S100β, a second commonly used marker for astrocytes [28]. The addition of the anti- S100β antibody did not increase the percentage of GFP^+^ cells identified as astrocytes compared to our original analysis using only the anti-GFAP antibody (at 1.5 wpi 3/347 and 2/376 co-localized with the S100β+GFAP stain in II-Cre or III-Cre infected mice respectively; at 3 wpi only 1/183 and 3/411 co-localized with the S100β+GFAP stain in II-Cre or III-Cre infected mice respectively). Additionally, to verify that we were not missing parasite-astrocyte interactions in non-GFP^+^ cells, we used confocal microscopy to analyze 0.5 and 1.5 wpi brain sections stained with anti-astrocyte and anti-*Toxoplasma* antibodies; we found no co-localization of parasite and astrocyte stains (N = 2 mice/time point/*Toxoplasma* strain, 9 sections/mouse). For oligodendrocytes, we stained sections with antibodies for oligodendrocytes and then used confocal microscopy to analyze the colocalization between GFP and the anti-oligodendrocyte stain. For II-Cre infected mice, oligodendrocytes accounted for 1–2% of GFP^+^ cells at 1.5 and 3 wpi (S2 Fig). For III-Cre infected mice, oligodendrocytes accounted for 0–2% at 1.5 and 3 wpi (S2 Fig).

Notably, at 1.5 wpi, in II-Cre infected tissue sections, ~40% of the GFP^+^ cells had an immune cell morphology and co-localized with stains for T-cells or macrophages/microglia (S2 Fig). At 3 wpi in II-Cre infected tissue and both time points in III-Cre infected tissue, less than 10% of the GFP^+^ cells co-localized with stains for T-cells or macrophages/ microglia (S2 Fig). As the goal of this work was to understand parenchymal CNS cell-*Toxoplasma* interactions, further studies on the immune cells were not pursued.

Finally, by 3 wpi, in both II-Cre and III-Cre infected mice, the majority of the unidentified GFP^+^ cells were morphologically neurons but did not stain with our anti-neuronal antibodies (Fig 1a red arrows, S1a and S1d Fig red arrows). We speculate that these GFP^+^ neurons did not stain with the neuronal antibodies because they are under-going cell death or have lost antigenicity in response to stress [29]. Of equal importance, unidentified cells never displayed an astrocytic morphology. While astrocyte morphology can vary across the brain, in the neuroanatomic regions we most commonly examined and during inflammatory states, the morphology is most often stellate in shape (S1b Fig) [28,30].

Together, these data suggest that throughout CNS infection and regardless of infecting strain, neurons make up the majority of the parasite-injected parenchymal CNS cells.

### IFN-γ depletion leads to an increase in parasite burden, GFP^+^ cells, and GFP^+^ astrocytes

Given that astrocytes are readily and even preferentially invaded *in vitro* [10], we were surprised that they represented such a low percentage of parasite-injected CNS cells *in vivo*. As such, we sought to determine the mechanisms involved in this unexpected *in vivo* tropism. We hypothesized that IFN-γ might play a critical role in influencing CNS cell-parasite interactions because of its central role in controlling CNS toxoplasmosis [17]; its pleiotropic effects on cells [31], including enabling cells to kill intracellular parasites [18,20,21]; and its potential role in inducing parasites to switch from the actively replicating and disseminating tachyzoite to the slowly-replicating, persistent bradyzoite [32]. We were especially intrigued by this possibility because *in vitro* studies have shown that IFN-γ-inducible, immunity-related GTPases (IRGs) are a major mechanism by which murine cells–including astrocytes–kill intracellular parasites [18,20,33]. In addition, in murine embryonic fibroblasts and bone-marrow derived macrophages, IRG-deployment leads to intracellular parasite death, followed by host cell necrosis [34]. Given these data, we hypothesized that IFN-γ-stimulated astrocytes might become infected, kill the intracellular parasites, and die all prior to the expression of appreciable amounts of GFP, circumventing our ability to detect these transient interactions.

**Fig 2:**
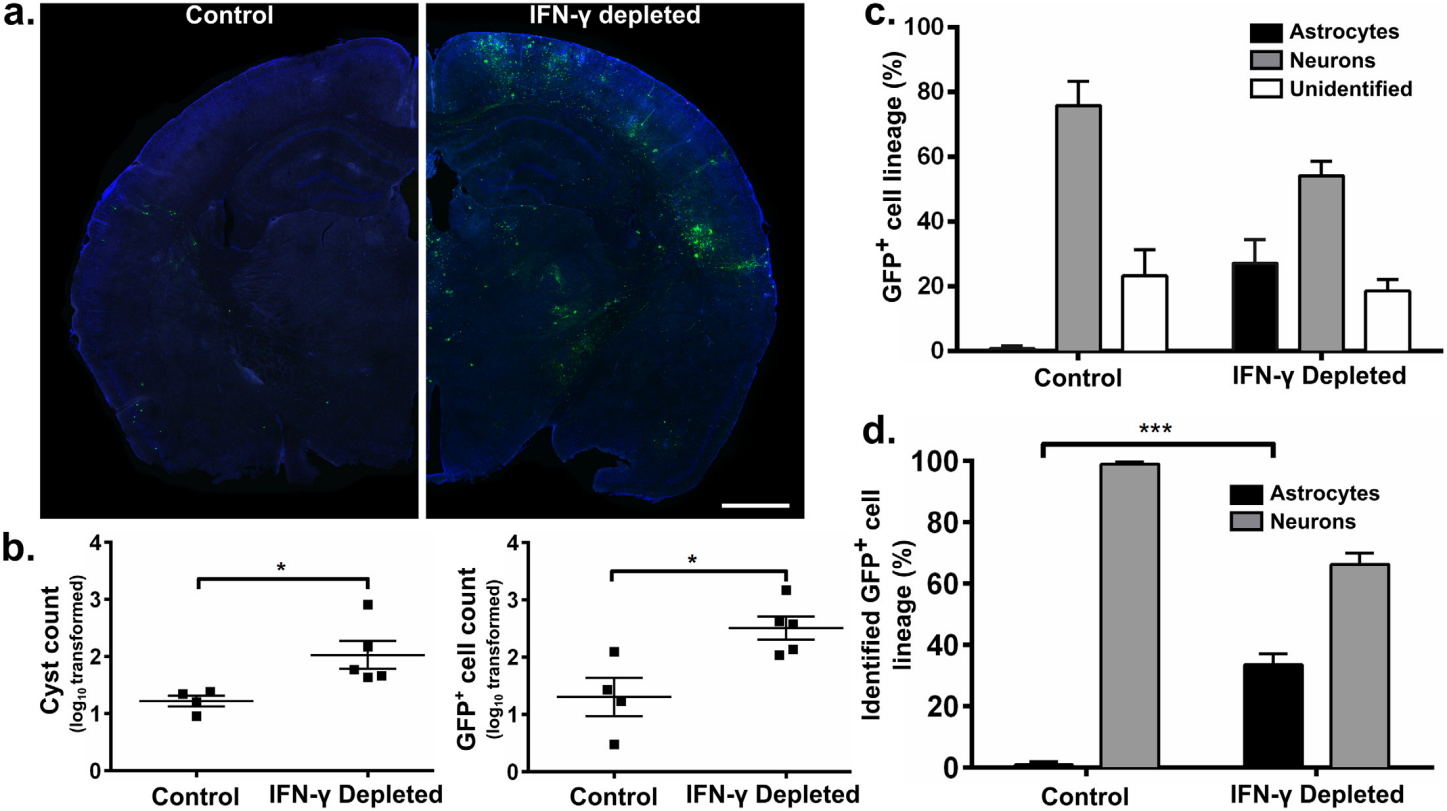
In the setting of IFN-γ depletion, *Toxoplasma* reactivation leads to an increase in parasite burden, GFP^+^ cells, and GFP^+^ astrocytes in the CNS. Starting at 4 wpi, isotype control or anti-interferon-γ antibodies were administered every 5 days to Cre reporter mice infected with II-Cre parasites. Mice were sacrificed at 6 wpi after receiving 3 doses of antibody treatment. Brains were sectioned and stained as in Fig 1. Confocal microscopy was used to analyze stained brain sections from control and IFN-γ-depleted mice. (a) Representative stitched-grid image of half of a coronal brain section from a control (left) or IFN-γ-depleted (right) mouse. Scale bar, 1 mm. (b) Quantification of cyst number (6 sections/mouse) and GFP^+^ cell number (1 section/mouse) found in control or IFN-γ-depleted mice. N = 4–5 mice/group. *p< 0.05 by independent sample, two-tailed t-test. (c) Quantification of the lineage of GFP^+^ cells by co-localization with antibody stains for neurons, astrocytes, or neither (unidentified). (d) As in (c) but restricting the analysis only to GFP^+^ cells identified as neurons or astrocytes. N = 4–5 mice/group. N = 82–209 GFP^+^ cells/mouse analyzed for cell lineage studies. ***p< 0.001 by independent sample, two-tailed t-test.

To test this hypothesis, we administered anti-IFN-γ or control, non-cytokine depleting, antibody to Cre reporter mice chronically infected with II-Cre parasites. After two weeks of antibody administration, brains were harvested, sectioned, stained, and analyzed as described in the preceding section. Serum taken at the time of sacrifice was tested for levels of IFN-γ. As expected, mice treated with anti-IFN-γ antibody showed a significant decrease in serum IFN-γ levels compared to mice treated with control antibody (S3 Fig). Brain sections from IFN-γ-depleted mice showed a marked increase in GFP^+^ cells compared to brain sections from control mice (Fig 2a). Quantification of CNS parasite cyst burden and total GFP^+^ cells revealed that IFN-γ-depleted mice had a ~ 10-fold increase in both parameters compared to control mice (Fig 2b). In IFN-γ-depleted mice, astrocytes now accounted for 27 ±7% of the GFP^+^ cells as opposed to 1 ±1% in control mice (Fig 2c). Neurons accounted for 54 ±4% of GFP^+^ cells in IFN-γ-depleted mice and 76 ±8% in control mice, with the remaining cells classified as unidentified (19 ±3% in IFN-γ depleted mice and 23 ±8% in control mice) (Fig 2c). Even in this extreme situation of unrestricted parasite growth, GFP^+^ neurons still outnumber GFP^+^ astrocytes almost 2:1 (66 ±4% vs. 34 ±4%, Fig 2d). Together these data suggest that IFN-γ plays a role in CNS host cell interactions but even when IFN-γ is depleted neuron-parasite interactions predominate.

### IRG-dependent astrocyte death does not account for lack of GFP^+^ astrocytes

While the above experiments demonstrate that IFN-γ-depletion affects CNS host cell interactions, they do not define the mechanism. The increase in GFP^+^ astrocytes could be explained either by i) enabling our detection of parasite-astrocyte interactions that routinely occur but could not be observed (i.e. blocking IRG-dependent host cell necrosis) or by ii) uncontrolled parasite growth leading to an increased frequency in parasite-astrocyte interactions. To distinguish between these two possibilities, we took advantage of the finding that cells infected with IRG-resistant parasites do not undergo IRG-dependent host cell death [34]. We reasoned that if IRG-dependent astrocyte necrosis played a significant role in preventing our observation of astrocyte-parasite interactions in immunocompetent mice, then infection with IRG-resistant parasites should result in a significant increase in GFP^+^ astrocytes, mimicking what we observed with IFN-γ-depletion.

**Fig 3:**
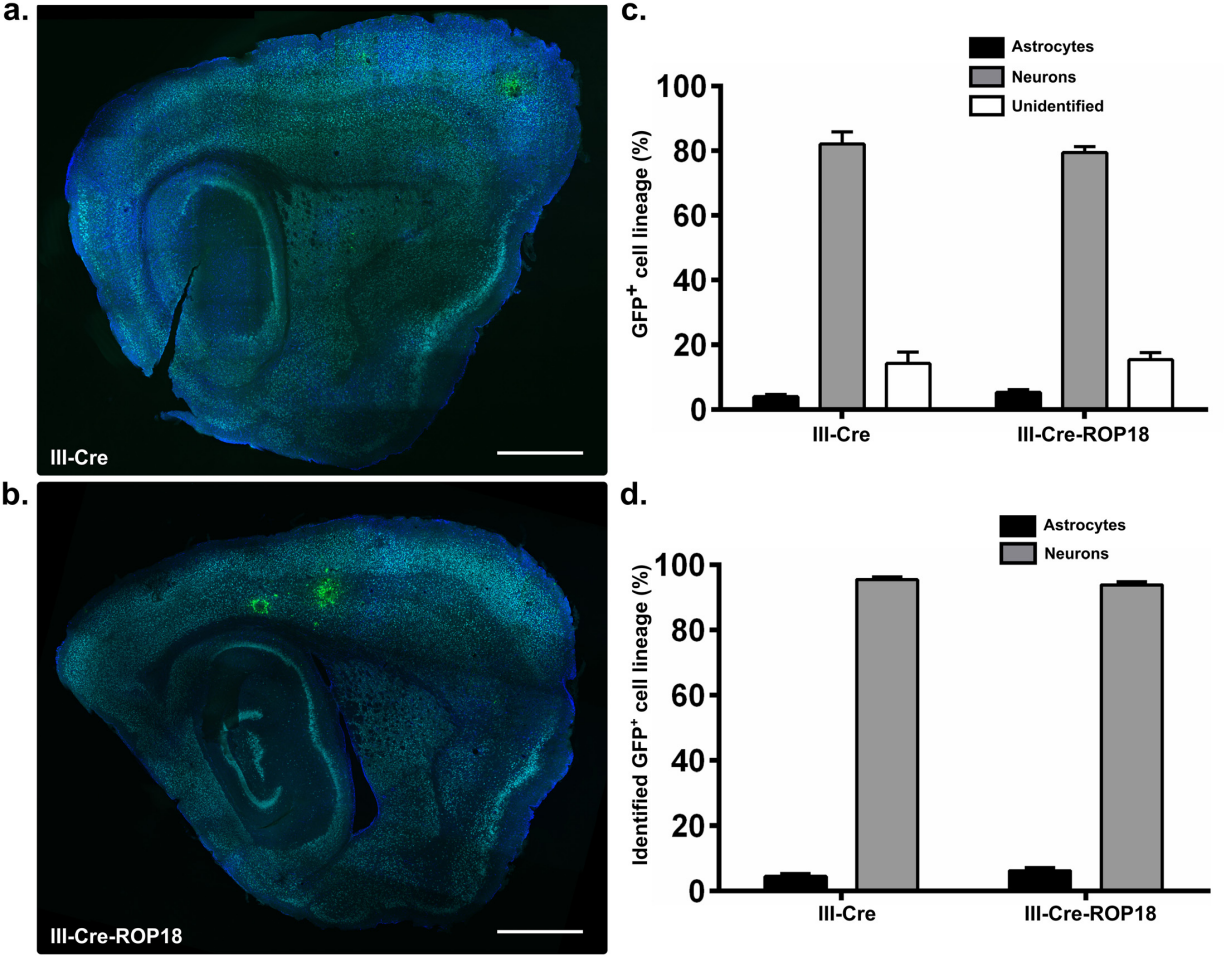
Neuron-parasite interactions predominate during infection with IRG-resistant *Toxoplasma* parasites. Cre reporter mice were infected with either III-Cre or III-Cre parasites that express the type I ROP18 protein (III-Cre-ROP18). At 2 weeks post infection (wpi), brains were harvested, sectioned, stained for astrocytes and neurons, and analyzed by confocal microscopy as in Fig 1. (a), (b) Representative merged stitched-grid image of a brain section from a III-Cre (a) or III-Cre-ROP18 (b)-infected mouse. Blue = astrocyte stain (GFAP), Cyan = neuronal stain (neuronal cocktail), Green = GFP expression. Scale bar, 1 mm. (c) Quantification of the lineage of GFP^+^ cells by co-localization with antibody stains for neurons, astrocytes, or neither. N = 100–114 GFP^+^ cells/mouse, N = 5 mice/*Toxoplasma* strain. (d) As in (c) but restricting the analysis only to GFP^+^ cells identified as neurons or astrocytes. There is no significant difference between the mean percentage of GFP^+^ neurons or astrocytes in III-Cre vs. III-Cre-ROP18 brain sections (p-value = 0.25, independent sample, two-tailed t-test.)

To engineer IRG-resistant parasites, we utilized the recent discovery that specific alleles of the parasite genes for ROP5 and ROP18 confer IRG-resistance [35,36]. The type III strain encodes for resistant alleles of ROP5, but because of an insertion just upstream of the ROP18 start codon, it expresses relatively little ROP18 [37,38], rendering the strain both IRG-sensitive and avirulent. By engineering a type III *Toxoplasma* strain to express the virulent type I ROP18 [39] as well as the Cre fusion protein, we created an encysting, IRG-resistant strain capable of triggering Cre-mediated recombination. For simplicity, we refer to this strain as III-Cre-ROP18. Consistent with previous reports [37,39], the expression of the type I ROP18 in the type III background made the III-Cre-ROP18 hypervirulent during acute infection (6/6 mice died < 3 wpi. Mice were inoculated with 100, 1000, or 10000 tachyzoites of III-Cre-ROP18, 2 mice/dosage). To ensure survival to the chronic stage of infection, after infecting Cre reporter mice with III-Cre-ROP18 parasites, the mice were treated with sulfadiazine during acute infection (5–11 dpi). Control mice were infected with the avirulent III-Cre parasites and received the same sulfadiazine treatment. We harvested, sectioned, and stained brains at 2 wpi. Stained brain sections were then analyzed by confocal microscopy for co-localization of GFP with neuronal or astrocytic staining. The III-Cre- and III-Cre-ROP18-infected mice had grossly similar CNS infections (Fig 3a and 3b). With both infecting strains, the vast majority of GFP^+^ cells were neurons (Fig 3c and 3d), with neurons making up 95 ±1% of identified GFP^+^ cells in III-Cre-infected mice and 94 ±1% of identified GFP^+^ cells in III-Cre-ROP18-infected mice (Fig 3d).

These data are consistent with the original wild-type data (Fig 1d) and disparate from the IFN-γ-depletion data (Fig 2d), suggesting that IRG-dependent parasite and astrocyte death do not explain the lack of GFP^+^ astrocytes in mice with an intact IFN-γ response. Instead, the data are consistent with an increase in the frequency of astrocyte-parasite interactions in the setting of IFN-γ-depletion.

### *Toxoplasma*-Cre parasites primarily interact with neurons when directly injected into the CNS of uninfected mice

Given this striking predominance of parasite-neuron interactions throughout CNS infection, we wondered what mechanisms might drive this interaction, especially as *Toxoplasma* readily invades both astrocytes and neurons *in vitro* [10–12,40]. As GFP^+^ cells were commonly found in the neocortex, we hypothesized that the location of CNS entry or reactivation may place parasites in neuroanatomic areas with a paucity of astrocytes, offering another explanation for our observation of few parasite-astrocyte interactions. This possibility was particularly appealing given that astrocyte density can vary widely over the CNS [28].

**Fig 4:**
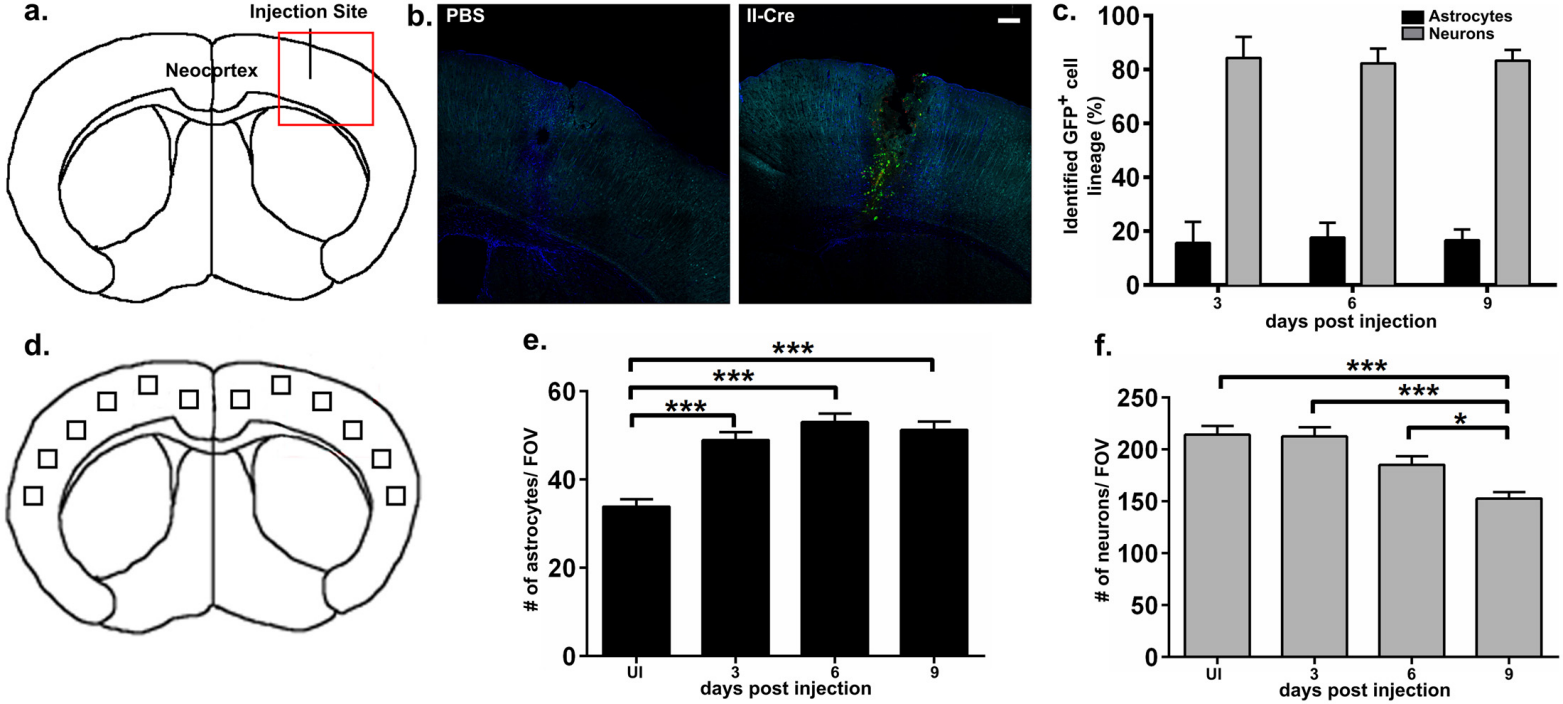
Neuron-parasite interactions dominate in direct CNS inoculation with *Toxoplasma*, consistent with the baseline ratio of neurons:astrocytes. Phosphate Buffer Saline (PBS) or *Toxoplasma*-Cre parasites (II-Cre) were stereotactically injected into the cerebral cortex of naïve Cre reporter mice. At 3, 6, and 9 days post-injection (dpi) brains were harvested, sectioned, stained for astrocytes and neurons, and analyzed by confocal microscopy for co-localization of GFP with astrocyte or neuronal stains or for baseline numbers of astrocytes and neurons. Brain sections from uninfected mice were also stained for neurons and astrocytes and analyzed for baseline numbers of astrocytes and neurons. (a) Schematic of site of injection. (b) Representative merged stitched-grid images of the injection site at 3 dpi from PBS (left) or *Toxoplasma*-Cre (right) injected mice. Blue = astrocyte stain (anti-GFAP), Cyan = neuronal stain (anti-neuronal cocktail), Green = GFP expression. Scale bar, 200 μm. (c) Quantification of GFP^+^ cells identified as either astrocytes or neurons by co-localization with staining. N = 3 mice/time point, 100 identified GFP^+^ cells/mouse. No statistical differences were found between the mean percentage of GFP^+^ neurons across time points (one-way ANOVA, p = 0.97). No GFP^+^ cells were seen in PBS injected mice. N = 1 mouse/time point. (d) Schematic of fields of view (FOV) taken to assess neuron and astrocyte numbers across IC infection time points. (e) Quantification of number of astrocytes/FOV. (f) Quantification of number of neurons/FOV. As astrocytes in uninfected mice express little GFAP (S4a Fig), these sections were stained with anti-GFAP, anti-S100β, and anti-ALDH1L1 antibodies. The neuronal cocktail was unchanged. UI, uninfected. N = 3–4 mice/time point, 3 randomly selected FOV/hemisphere/ mouse (6 FOV total/mouse) were counted. * p<0.05, *** p< 0.001, ordinary one-way ANOVA.

To test if location properties regulated which CNS cells interact with parasites, we inoculated II-Cre parasites directly into the neocortex of naïve Cre reporter mice. At 3, 6, and 9 days post inoculation (dpi), brains were harvested, sectioned, stained, and analyzed with confocal microscopy to categorize GFP^+^ cells as neurons, astrocytes, or unidentified. The percentage of GFP^+^ cells identified as neurons consistently outnumbered the percentage identified as astrocytes (82–84% vs. 16–18% respectively, Fig 4c), giving ratios of 4–5 GFP^+^ neurons for every GFP^+^ astrocytes. To determine if these ratios were consistent with the baseline number of neurons and astrocytes in the neocortex and throughout this infection, brain sections from the inoculated mice (including the PBS injected mice) and 3 uninfected mice were stained for astrocytes and neurons. From twelve fields of view (FOV) of the cortex (Fig 4d), we randomly selected 6 FOV (3 FOV/hemisphere) per mouse, and counted all cells identified as astrocytes (Fig 4e) or neurons (Fig 4f). The number of astrocytes/FOV was greater in 3^+^ dpi mice than in uninfected mice (Fig 4e, S4 Fig), which could be due to: i) the known increase in GFAP expression and therefore staining during CNS inflammation, ii) an increase in astrocyte number (proliferation), or iii) both. The increased visibility of astrocytic processes and lack of overlap of the processes or cell bodies at 3, 6, and 9 dpi (S4 Fig) is more consistent with GFAP up-regulation rather than proliferation [30]. Consistent with previous observations of loss of neuronal staining during *Toxoplasma* infection [41], the number of neurons/FOV significantly decreased by 9 dpi (Fig 4f, S4 Fig). As noted above, this loss of staining could be secondary to cell death or loss of antigenicity in response to stress [29]. Comparing the means between the total neuron and total astrocyte counts yielded neuron:astrocyte ratios of 3:1–6:1 (Fig 4e and 4f), which is consistent with the observed GFP^+^ neurons: GFP^+^ astrocytes ratios of 4:1–5:1 throughout CNS intracranial infection. Collectively, these data suggest that the ratio of neurons:astrocytes at the location of parasite entry could influence which CNS cells interact with parasites.

### *Toxoplasma* cysts are located primarily in neuronal processes

**Fig 5:**
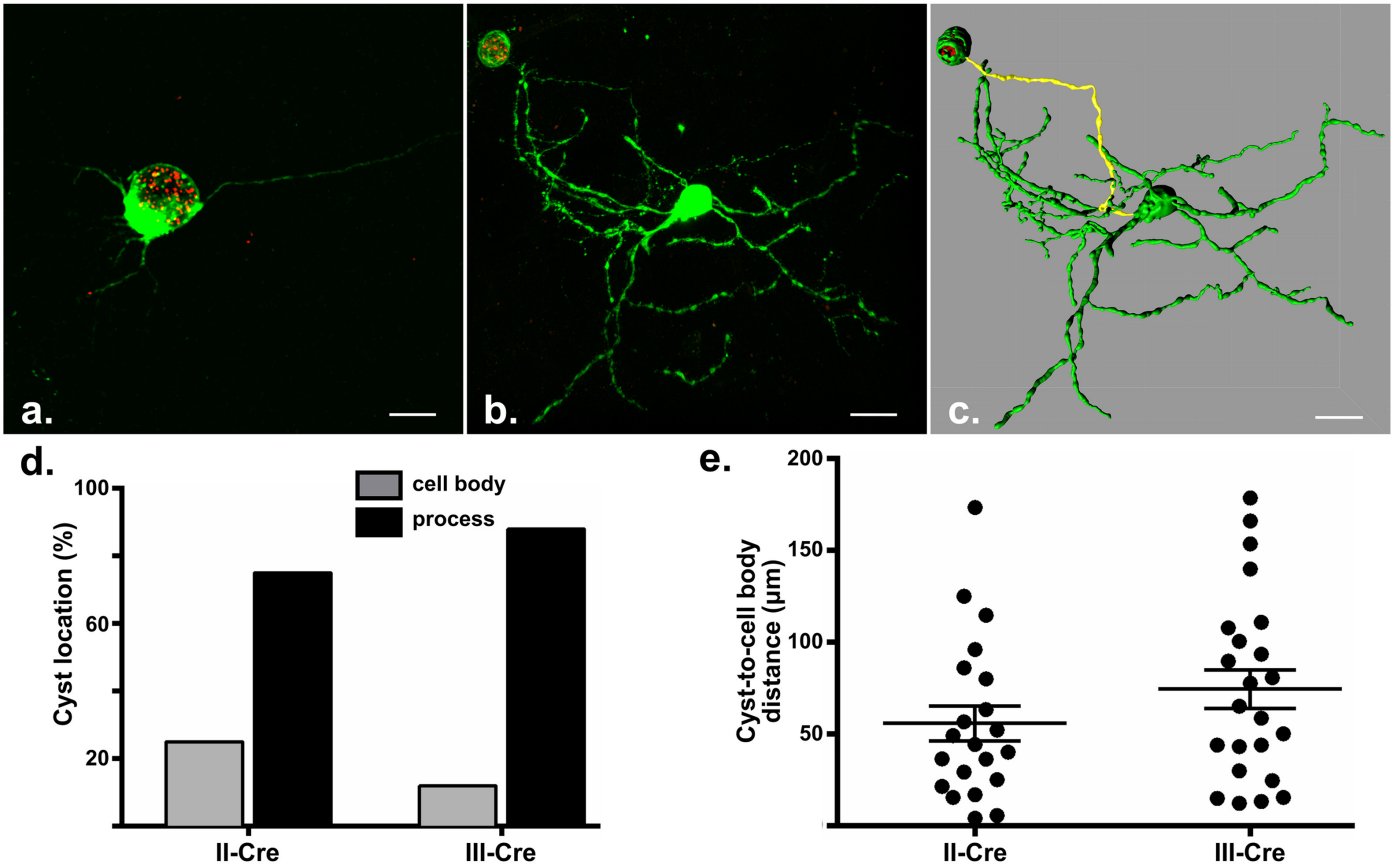
*Toxoplasma* cysts are primarily located in neuronal processes. Cre reporter mice were infected with II-Cre or III-Cre parasites. At 3 weeks post infection, brains were harvested, sectioned into ~200 μm thick sections, processed to render the tissue optically clear, and then imaged at 40x on a confocal microscope. Resulting images were then analyzed with Imaris software to identify the cellular location of the cyst (neuronal process or cell body). For cysts within a process, imaging analysis software was used to determine the distance between the cyst and the cell body, along the infected process. (a) Representative maximal projection image of a cyst within a cell body. S1 Video shows a 3-D movie of this cell. (b) Representative maximal projection image of a cyst within a neuronal process. (c) Representative maximal projection image of (b) analyzed by Imaris to identify the whole neuron and to determine the length from the cyst edge to the edge of the cell body (yellow highlighted line). S2 Video shows a 3-D movie of this cell. Scale bars, 20 μm. (d) Quantification of the percentage of cysts found in the cell body or a neuronal process in mice infected with II-Cre or III-Cre parasites (as labelled). N = 26–28 cysts (III-Cre and II-Cre respectively). (e) Quantification of the distance between cysts and cell bodies for II-Cre and III-Cre cysts within neuronal processes. Cysts in the cell body (N = 7, II-Cre, N = 3, III-Cre) were excluded from this analysis. Each dot represents a single cyst-cell body measurement. Bars, mean ±SEM.

As other physical characteristics besides neuron and astrocyte density distinguish these cell types, we hypothesized that other neuronal factors besides location might also play a role in the exclusivity of the parasite-neuron interaction. One obvious possibility was the size or surface area of neurons, which includes extensive dendritic arbors and axons that can run the length of an animal. We hypothesized that this widespread network of neuronal processes might lead to parasites having a higher probability of interacting with and invading neurons rather than the more compact astrocyte (S1b Fig). If our hypothesis was plausible, then one would expect to find parasites primarily in neuronal processes rather than cell bodies. To determine the cellular location of parasites within neurons, we took advantage of our recent development of a protocol for imaging whole infected neurons *in situ* [42]. By using this technique to analyze II-Cre or III-Cre-infected brain sections, we were able to delineate if cysts were located in neuronal cell bodies (Fig 5a, S1 Video) or processes (Fig 5b) and determine the distance between the cyst and cell body (Fig 5c, S2 Video). Consistent with our hypothesis and regardless of infecting strain, this analysis revealed that cysts are most often found in neuronal processes (Fig 5d, 21/28 II-Cre cysts and 23/26 II-Cre cysts) and are commonly far from the cell body, with mean cyst-to-cell-body distance of 56 ±9 μm for II-Cre cysts and 75 ±10 μm for III-Cre cysts (Fig 5e). Given that cysts are predominantly found in neuronal processes and are often a significant distance from the cell body, these data suggest that neuronal size could also play a role in driving *Toxoplasma*’s predilection for neurons.

## Discussion

In this study, we used a novel *in vivo* system to address an essential question: why does *Toxoplasma gondii*, a parasite with no clear host cell preference *in vitro*, show host cell preferences during *in vivo* infection of the CNS? Our ability to answer this question arose from the development of the *Toxoplasma*-Cre system, which is capable of identifying transient host cell-parasite interactions and allows imaging and analysis of entire infected neurons [22,24,42]. The data derived from this system and presented here strongly suggest that neuron-parasite interactions predominate throughout CNS infection and persist even in highly manipulated circumstances such as IFN-γ depletion, infection with IRG-resistant parasites, and direct intracranial inoculation of parasites. These data in combination with the recognition that cysts are most commonly found in distal neuronal processes indicate that during *in vivo* infection, *Toxoplasma* displays a distinct propensity for neurons, which may in part be driven by physical properties of the CNS and its cells.

There are several important implications of these findings. Excitingly, the predominance of the neuron-parasite interaction implies that neurons are not mere bystanders that are incidentally infected by parasites, but rather that neurons are critical participants in the establishment of the persistent CNS infection, a concept consistent with a recent report showing that transgenic mice that lack gp130 in neurons are unable to control CNS toxoplasmosis [43]. In addition, the high number of uninfected GFP^+^ neurons [24] raises the tantalizing possibility that most neurons clear parasites by unrecognized means such as the recently described CD40-CD154 autophagy pathway [44,45]. An equally provocative explanation for the uninfected GFP^+^ neurons is that direct parasitic manipulation of these long-lived CNS cells without invasion plays an unrecognized role in promoting parasite survival and transmission. In either case, future work will focus on determining the origins of the GFP^+^ uninfected neurons and how these neurons differ from actively infected neurons or neurons with no direct parasite interactions.

Interestingly, our data from the intracranial injection experiments and our cyst location profiling suggest that physical properties of the CNS may play a role in driving this *Toxoplasma*-neuron predilection. As *in vitro* cultures cannot approximate the 3D nature of the *in vivo* CNS, these physical properties provide an appealing explanation for the discrepancy between prior *in vitro* work and our *in vivo* work. Of course, caution must be exercised when extrapolating from either set of experiments. The intracranial injection route is unnatural and skips the preceding systemic infection which provides cytokines that would be predicted to change the CNS environment even prior to parasite entry. The cyst location studies were done at a single time point, and do not address whether parasites invade and encyst in the same location or rather invade in one part of the neuron and migrate to the processes. Future work will focus on identifying the physical environment faced by parasites during entry into the CNS and how parasite location in the neuron changes from the time of entry to encystment.

Of equal importance is the limited evidence we found for direct interactions between parasites and astrocytes. As noted before, the current understanding of astrocyte-*Toxoplasma* interactions is derived from *in vitro* work, and suggests that cysts are not found *in vivo* in astrocytes [15,46] because astrocytes kill the intracellular parasites that readily invade this cell type [18,20,21,33]. This “astrocyte-killing” model would predict that using our *Toxoplasma*-Cre system we should find many uninfected GFP^+^ astrocytes, but instead we observed few. The findings presented here offer a different explanation: cysts are seldom found in astrocytes because parasites rarely directly interact with astrocytes in hosts with intact IFN-γ responses. In addition, the lack of GFP^+^ astrocytes could be considered to indirectly support the possibility of parasites entering into the CNS via immune cell carriers [26,47]. If parasites directly enter the CNS from the vasculature (either paracellularly or through infection of endothelial cells and egress from the basolateral side of the endothelial cell), parasites should next encounter astrocytes and the astrocytic endfeet that surround the vasculature [30]. Thus, to interact predominantly with neurons, parasites would have to migrate beyond the vasculature-associated astrocytes. On the other hand, if immune cells carry parasites into the CNS, parasite egress might only occur after infected immune cells pass these astrocytic processes.

Of course, it is difficult to prove the non-existence of an interaction. Our system would not detect direct astrocyte-parasite interactions that occurred without the injection of parasite proteins, though such a phenomenon has yet to be described or predicted. Additionally, as noted above, our system would not detect direct astrocyte-parasite interactions if infected astrocytes died prior to the expression of GFP. We think astrocyte death is unlikely to account for the lack of evidence for direct astrocyte-parasite interactions for several reasons. First, while several classes of IFN-γ-dependent GTPases are used by astrocytes to kill intracellular parasites [20,21], only IRG-deployment has been linked to subsequent death of the host cell *in vitro* [34]. We directly addressed this concern by using IRG-resistant III-Cre-ROP18 parasites and found no increase in the percentage of GFP^+^ cells identified as astrocytes (Fig 3). Second, given that we and others have reported significant numbers of GFP^+^ uninfected cells *in vivo* in immune cells [24,25,48] as well as neurons, rapid astrocyte death after parasite interaction would require invoking a unique mechanism of death that presumptively does not occur in these other cell types. Third, a recent report found no evidence for significant astrocytic death during CNS toxoplasmosis [41], further decreasing the likelihood that astrocyte death accounts for the paucity of GFP^+^ astrocytes.

Thus, given the current lack of evidence for either parasite invasion without injection of parasite proteins or astrocytic death during CNS toxoplasmosis, these data suggest that astrocytes primarily constrain *Toxoplasma* growth through indirect mechanisms such as barrier formation and/or secretion of cytokines and chemokines [30], rather than by direct killing of parasites. This possibility is consistent with prior work showing that mice lacking the glial fibrillary acid protein (GFAP) or the gp130 receptor only in astrocytes–two defects that would not be expected to impact astrocytic killing of intracellular parasites–develop uncontrolled CNS toxoplasmic encephalitis [49,50]. Additionally, the IFN-γ depletion experiments suggest that in severely immunocompromised patients, such as AIDS patients [51], an increase in direct parasite-astrocyte interactions may play an unrecognized role in the neuropathology of toxoplasmic encephalitis. Together, these findings offer new opportunities to dissect out the changing role of astrocytes in health and disease.

In conclusion, the findings presented here strongly suggest a new paradigm for CNS-*Toxoplasma* interactions. In this new paradigm, direct neuron-*Toxoplasma* interactions play an active and central role in the establishment of a successful, chronic CNS infection.

## Materials and Methods

### Ethics statement

All mouse studies and breeding were carried out in strict accordance with the Public Health Service Policy on Human Care and Use of Laboratory Animals. The protocol was approved by the University of Arizona Institutional Animal Care and Use Committee (#A-3248-01, protocol #12–391).

### Parasite maintenance

All strains were maintained through serial passage in human foreskin fibroblasts (gift John Boothroyd, Stanford University, Stanford, CA) using DMEM, supplemented with 10% fetal bovine serum, 2mM glutagro, and 100 I.U./ml penicillin/ 100 μg/ml streptomycin. In all experiments, except for those involving the type III-Cre-ROP18 strain, previously described type II (Prugniaud) and type III (CEP) strains that express Cre and mCherry were used [24,25]. To engineer the type III strain that expresses a type I ROP18, previously described molecular techniques were used [22], with parasites co-transfected with the *pToxofilin-Cre* plasmid [22] and a plasmid that contained an expression cassette for the type I ROP18 with the endogenous 5’ UTR and an expression cassette for the fluorescent protein mCherry flanked by the *GRA2* promoter and 5’ UTR and *GRA2* 3’-UTR [24]. The original *ROP18* expression plasmid [52] (gift Jeroen Saeij, UC, Davis, CA) was modified by standard molecular techniques to remove the *HPT* expression cassette and add the mCherry expression cassette, resulting in the *pROP18*_*I*_ *mCherry* plasmid. To verify secretion of a functional toxofilin:Cre fusion protein single cell clones were tested for efficacy in causing Cre-mediated recombination in Cre-reporter cells [22]. Increased virulence of the III-Cre-ROP18 strain (now LD_100_ with inoculum of only 50 parasites) was considered evidence for expression of a functional type I ROP18.

### Mice

All mice used in this study are Cre reporter mice that only express GFP in their cells after Cre-mediated recombination [23]. Mice were purchased from Jackson Laboratories (stock # 007906) and bred in the University of Arizona, BIO5 Animal Facility. Unless otherwise specified, mice were inoculated intraperitoneally (i.p.) with freshly syringe-released parasites, diluted to the appropriate inoculums in 200 μl volume in USP grade PBS. The inoculating number of parasites was 10,000 (II-Cre) or 100,000 (III-Cre) except where otherwise specified.

### *In vivo* IFN-γ depletion experiments

At 4 weeks post infection (wpi), mice infected with II-Cre parasites were treated i.p. with either 2 mg of anti-IFN-γ antibody (clone XMG1.2; BioXcell, BE0055) or rat IgG1 isotype-matched control antibody (BioXcell; BE0088). Two additional doses of either anti-IFN-γ antibody or Rat IgG1 isotype-matched control antibody were administered every 5 days. Two days post administration of the third dose mice were sacrificed and brains and blood were harvested. Serum IFN-γ levels were measured using a mouse IFN-γ ELISA kit (eBioscience, BMS606INST), and following the manufacturer’s recommended protocol.

### Experiments requiring sulfadiazine treatment

Mice were infected i.p. with freshly syringe-released parasites, III-Cre (10,000 tachyzoites) or III-Cre-ROP18 (10,000 tachyzoites), in 200 μl of USP grade PBS. Starting at 5 days post infection (dpi), mice received sulfadiazine sodium salt in their water (100mg/L) (S6387-25G; Sigma Aldrich). At 11 dpi, sulfadiazine-containing water was replaced by fresh water. The mice were sacrificed at 2 wpi and their brains were harvested.

### Intracranial infection experiments

For *in vivo* intracranial infection, Cre-reporter mice were anesthetized via nose cone (Stoelting Mouse Gas Anesthesia Mask; 51609) with isofluorane gas. The surgical site was shaved and cleaned, lidocaine jelly was applied to the site and buprenorphine (0.1mg/kg) was administered subcutaneously. The mouse was then placed into a stereotaxic device (Stoelting Just for Mice Stereotaxic Instrument; 51730), on a heating pad, and a midline incision was made to expose the skull. The skull was cleaned and dried with sterile cotton tipped applicators. Using the following stereotaxic coordinates: 2.0mm lateral and 0.25mm anterior relative to the bregma, a 23 gauge needle was used to bore a hole into the skull. One thousand freshly syringe-released II-Cre parasites diluted in 1 μl of USP grade PBS were loaded into a syringe (Hamilton; 7634–01, 7803–05) and injected at the stereotaxic coordinates and 1.5 mm deep using a Stoelting Quintessential Stereotaxic Injector (53311) at a rate of 0.5μl/min. After recovery, mice were closely monitored until the brains were harvested at 3, 6 and 9 dpi.

### Cyst location experiments

At 3 wpi, brains from infected Cre-reporter mice were harvested, sectioned, optically cleared and imaged as previously described [42]. In brief, ~200 μm thick brain sections were cut on a Vibratome (Series 1000) and stored in cryoprotective media (see tissue preparation) until processing. To optically clear, sections were rinsed in PBS then placed into a vial of 25% glycerol/pbs-tween-20 (PBST) solution and kept covered from light, on an orbital shaker at 4°C until they sunk to the bottom of the vial. After sinking, sections were changed into 50%, 75% and 90% solutions respectively for 12–24hrs each. After clearing, sections were mounted onto slides with spacers in 90% glycerol/PBST solution. Z-stack images were obtained by confocal microscopy through sections to include whole cells containing cysts. Imaris software was used to visualize, detect, render and measure lengths of processes harboring cysts.

### Tissue preparation

For immunohistochemical analyses, on the appropriate day or week post infection, mouse brain tissue was harvested, fixed, and sucrose embedded as previously described [24]. Forty micron thick sagittal or coronal brain sections were cut on a freezing sliding microtome (Microm HM 430) and stored as free floating sections in cryoprotective media (0.05 M sodium phosphate buffer containing 30% glycerol and 30% ethylene glycol) until stained and mounted on slides.

### Immunofluorescence Assays

For immunofluorescence, brain sections were stained with the following primary antibodies: **Neurons**: biotin conjugated anti-NeuN-B clone A60 (MAB377; Millipore, 1:200), mouse anti-MAP2 (2a+2b) (M2320; Sigma-Aldrich, 1:2000), chicken anti-200 kD Neurofilament Heavy antibody (ab4680; Abcam 1:20,000). This cocktail was used for all figures. **Astrocytes**: rabbit anti-glial fibrillary acidic protein (GFAP) (Z0334; Dako, 1:200) was used for all Figures except for Fig 4e, S4a Fig where rabbit anti-S100 (Z0311; Dako, 1:200) and rabbit anti-ALDH1L1 (ab87117, Abcam, 1:500) were also used. **Oligodendrocytes**: goat anti-Olig2 (AF2418R&D systems, 1:500). **T-cells**: hamster anti-mouse CD3ε 5000A2 (550277, BD Pharmigen, 1:500). **Macrophages/ microglia**: chicken anti-Iba-1 (139590, Abcam, 1:2000). **Parasites:** mouse anti-SAG1 (gift John Boothroyd, 1:100,000); rabbit anti-SRS9 (gift John Boothroyd, 1:2000), and biotinylated Dolichos Biflorus Agglutinin (B-1035; Vector Laboratories Inc., 1:500). The following species-appropriate secondary antibodies were used: Alexa Fluor 405 goat anti-rabbit IgG, Alexa Fluor 555 goat anti-chicken IgG (1:500), Alexa Fluor 555 donkey anti-goat IgG, Cy-5 Streptavidin (Invitrogen), Alexa Fluor 647 goat anti-mouse IgG, Alexa Fluor 647 goat anti-chicken IgG, Alexa Fluor 647 goat anti-hamster IgG, and DyLight 405-conjugated Streptavidin (Jackson Immuno Research Laboratories, Inc. 1:2000). Unless otherwise noted, secondary antibodies were obtained from Life Technologies and used at a concentration of 1:200. Sections were mounted as previously described [24].

### Microscopy

An inverted Leica SP5-II resonant scanner confocal microscope (Leica Microsystems, Buffalo Grove, IL) with standard LAS AF software was used to generate images for co-localization studies, the cortical neuron and astrocyte counts, and high resolution stitched grid images. A Zeiss LSM 510 Meta confocal microscope with standard LSM software was used to generate images for cyst location studies. All images shown in a given figure and with a given color were obtained using identical parameters. Images were processed using ImageJ software, Imaris 7.6.5 software (Bitplane) and/or Adobe Photoshop. Cortical neuron and astrocyte counts were manually counted using the cell counter plug-in for Image J.

### Statistical analyses

All statistical analyses were performed using GraphPad Prism (version 6.0f, 1994–2014 GraphPad Software, Inc., La Jolla, CA). Statistical tests for any given set of data are described in the figure legend. In brief, for direct comparisons between two groups, an independent sample, two-tailed t-test was used. For cyst counts, absolute GFP^+^ cell counts, and serum IFN-γ levels in experiments with IFN-γ depletion, the counts were log_10_ transformed prior to graphing and subjection to t-test. In any experiment comparing multiple time points, one-way ANOVA testing was performed to determine if the mean percentage of a given GFP^+^ cell-type varied over time.

## Supporting Information

S1 Fig GFP^+^ cells are identified by co-localization with standard staining for different cell lineages. Forty micron brain sections are stained with antibodies against astrocyte proteins (anti-astrocyte, blue) and/or neuronal proteins (anti-neuron, cyan). Sections are then analyzed by confocal microscopy to identify co-localization of GFP^+^ cells and antibody staining. (a) Representative image of GFP^+^ cells with morphology consistent with neurons (big nuclei with large straight processes emanating from cell body) and that co-localizes with antibody staining for neurons (white arrowheads) or do not clearly co-localize with anti-neuronal cocktail antibodies (red arrowhead). As the GFP^+^ cell at which the red arrowhead points is not clearly co-localizing with anti-neuron antibody staining, it is considered “unidentified”. (b) Representative image of GFP^+^ cell with astrocyte morphology (nucleus surrounded by short, radiating processes) and that co-localize with astrocyte antibody staining (white arrowhead). (c) GFP^+^ cells with morphology most consistent with immune cells (small cells, no projections) (red arrowheads) and without co-localization with astrocyte or neuronal markers. (d) GFP^+^ cell with neuronal morphology but no co-localization with anti-astrocyte or neuron stains (red arrowhead). Scale bars, 20 μm. Images (a), (b), and (c) are from a II-Cre infected mouse at 3 wpi. Image (d) is from a III-Cre infected mouse at 2 wpi.
(TIF)

S2 Fig Few GFP^+^ cells are identified as oligodendrocytes. Brain sections from the same mice represented in Fig 1 were stained for oligodendrocytes (anti-Olig2) or T-cells (anti-CD3e) and macrophages/microglia (anti-Iba1). Stained sections were analyzed by confocal microscopy to identify GFP co-localization with described stains. Graphs show the percentage of GFP^+^ cells identified as oligodendrocytes, T-cells, or macrophages/microglia at specified time points for II-Cre (**left graph**) or III-Cre (**right graph**) infected mice. No GFP^+^ cells co-localized with Olig2 staining at 3 wpi in III-Cre infected mice. Bars, mean ±SEM. In II-Cre-infected mice, for oligodendrocytes, N = 62–111 GFP^+^ cells examined/ infected mouse, 3 mice/time point (total of 229–317 GFP^+^ cells evaluated/time point). For T-cells and macrophages/microglia, N = 57–163 GFP^+^ cells examined/ infected mouse, 3 mice/time point (total of 229–317 GFP^+^ cells evaluated/ time point.) In III-Cre-infected mice, for oligodendrocytes, N = 100–128 GFP^+^ cells examined/ infected mouse, 4 mice/time point, (total of 438–447 GFP^+^ cells evaluated/time point.) For T-cells and macrophage/microglia, N = 100–163 GFP+ cells examined/ infected mouse, 4 mice/time point, (total of 432–520 GFP^+^ cells per time point.)
(TIF)

S3 Fig Mice treated with IFN-γ antibody show a significant decrease in serum IFN-γ levels compared to control mice treated with non-depleting antibody. Serum IFN-γ levels determined by specific ELISA. N = 4–5 mice/ treatment. Bars, mean ±SEM. ***p< 0.001 by independent sample, two-tailed t-test.
(TIF)

S4 Fig Astrocyte immunofluorescence staining is greater in infected mice than uninfected mice. Forty micron brain sections are stained with antibodies against astrocyte proteins (anti-astrocyte) and/or neuronal proteins (anti-neuron). Representative inverted-color, maximal projection images from 8 μm stack of cells stained with (a) anti-neuron or (b) anti-astrocyte stains or (c) with GFP expression at labeled time points. As astrocytes in uninfected mice express little GFAP, brain sections analyzed for these purposes were stained with anti-GFAP, anti-S100β, and anti-ALDL1H1. Anti-S100β stains astrocytic nuclei/cytoplasm, anti-ALDL1H1 stains astrocytic cytoplasm, and anti-GFAP stains astrocytic processes. Note that for astrocytes, progressing from uninfected to 9 dpi, processes that stain with anti-GFAP antibodies are more clearly identified but still do not overlap in space. Scale bar, 50 μm.
(TIF)

S5 Fig Schematics of brain sections. (a) Schematic of sagittal brain section shown in Fig 1a. (b) Schematic of coronal brain section(s) shown in Fig 2a. Whole brain section is drawn here while Fig 2a shows two hemi-sections placed next to each other. (c) Schematics of sagittal brain sections shown in Fig 3a and 3b. Major brain areas are labeled. H = hippocampus. Gray shading represents the corpus collosum, a major white matter tract. Black shading represents ventricular space filled with cerebrospinal fluid.
(TIF)

S6 Fig Montage of individual z-stack images showing the GFP fully surrounding the cyst in S1 Video. (TIF)

S1 Video 3-D rendering of *Toxoplasma* cyst located inside cell body. Imaris 7.6.5 software was used to create a 3-D movie from z-stack images through the II-Cre infected neuron in Fig 5a. After detection of the neuron cell body, neuronal processes and the cyst, a surface rendering is created and the background is removed. Full rotation of the z-stack reveals that the entire cyst is located within the cell body. Areas of the cell have been stretched so thin that the Imaris software is unable to detect the limited concentration of GFP in these areas, which creates a window-like appearance. On individual z-stack images, GFP can be found fully surrounding the cyst (S6 Fig).
(MP4)

S2 Video 3-D rendering of *Toxoplasma* cyst located in distal neuronal process. 3-D movie created as in S2 Video but from z-stack images through the III-Cre infected neuron in Fig 5b. Rotation of the z-stack allows for full visualization and measurement of the process harboring the cyst (yellow).
(MP4)
