# Supplementary figures and images for: Neurons are the Primary Target Cell for the Brain-Tropic Intracellular Parasite Toxoplasma gondii
Source: PLoS Pathog. 2016 Feb 19;12(2):e1005447. doi: 10.1371/journal.ppat.1005447 (PMC4760770; doi:10.1371/journal.ppat.1005447)

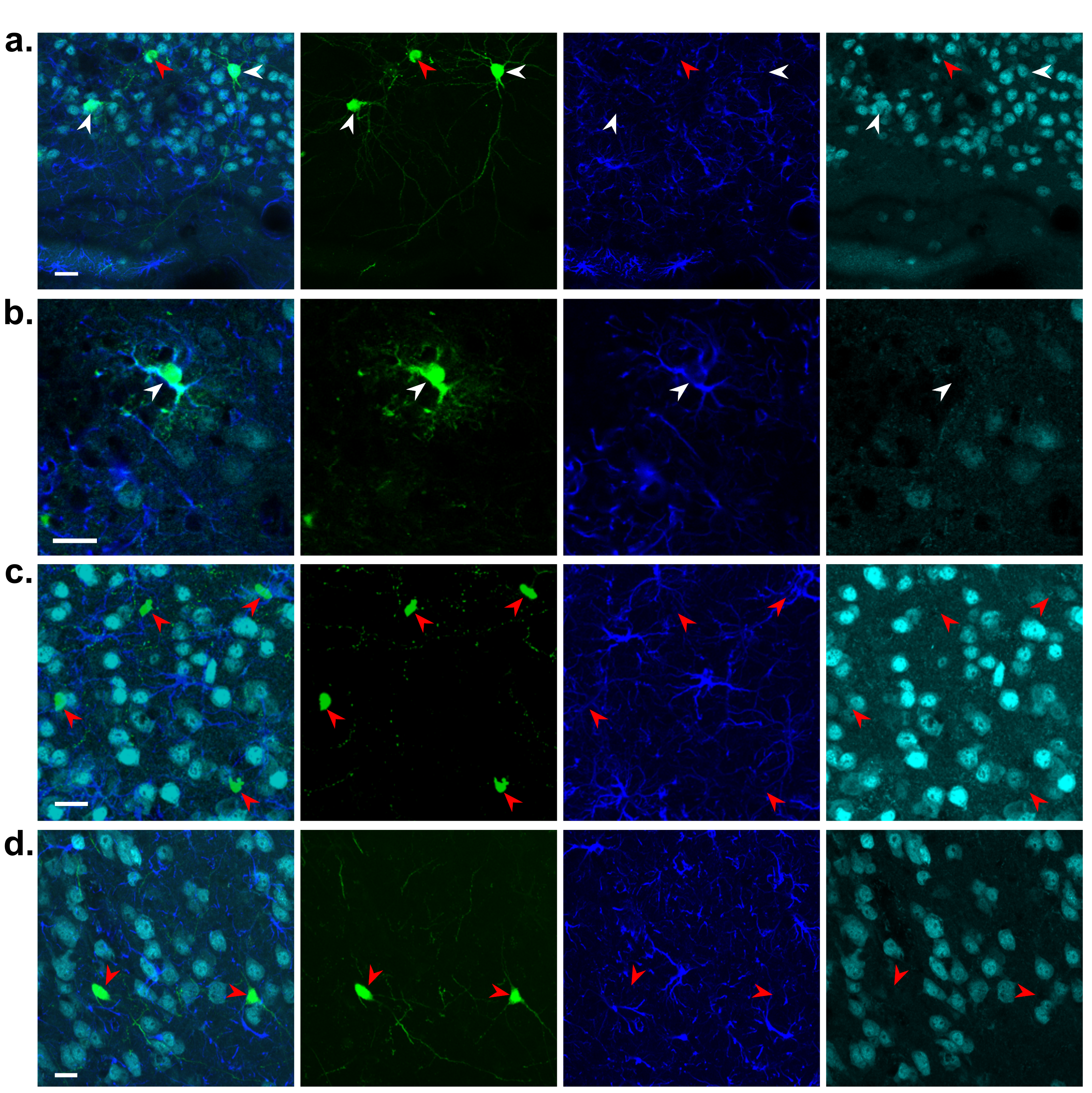

Supplement: S1 Fig — Forty micron brain sections are stained with antibodies against astrocyte proteins (anti-astrocyte, blue) and/or neuronal proteins (anti-neuron, cyan). Sections are then analyzed by confocal microscopy to identify co-localization of GFP+ cells and antibody staining. (a) Representative image of GFP+ cells with morphology consistent with neurons (big nuclei with large straight processes emanating from cell body) and that co-localizes with antibody staining for neurons (white arrowheads) or do not clearly co-localize with anti-neuronal cocktail antibodies (red arrowhead). As the GFP+ cell at which the red arrowhead points is not clearly co-localizing with anti-neuron antibody staining, it is considered “unidentified”. (b) Representative image of GFP+ cell with astrocyte morphology (nucleus surrounded by short, radiating processes) and that co-localize with astrocyte antibody staining (white arrowhead). (c) GFP+ cells with morphology most consistent with immune cells (small cells, no projections) (red arrowheads) and without co-localization with astrocyte or neuronal markers. (d) GFP+ cell with neuronal morphology but no co-localization with anti-astrocyte or neuron stains (red arrowhead). Scale bars, 20 μm. Images (a), (b), and (c) are from a II-Cre infected mouse at 3 wpi. Image (d) is from a III-Cre infected mouse at 2 wpi. (TIF) [file ppat.1005447.s001.tif]

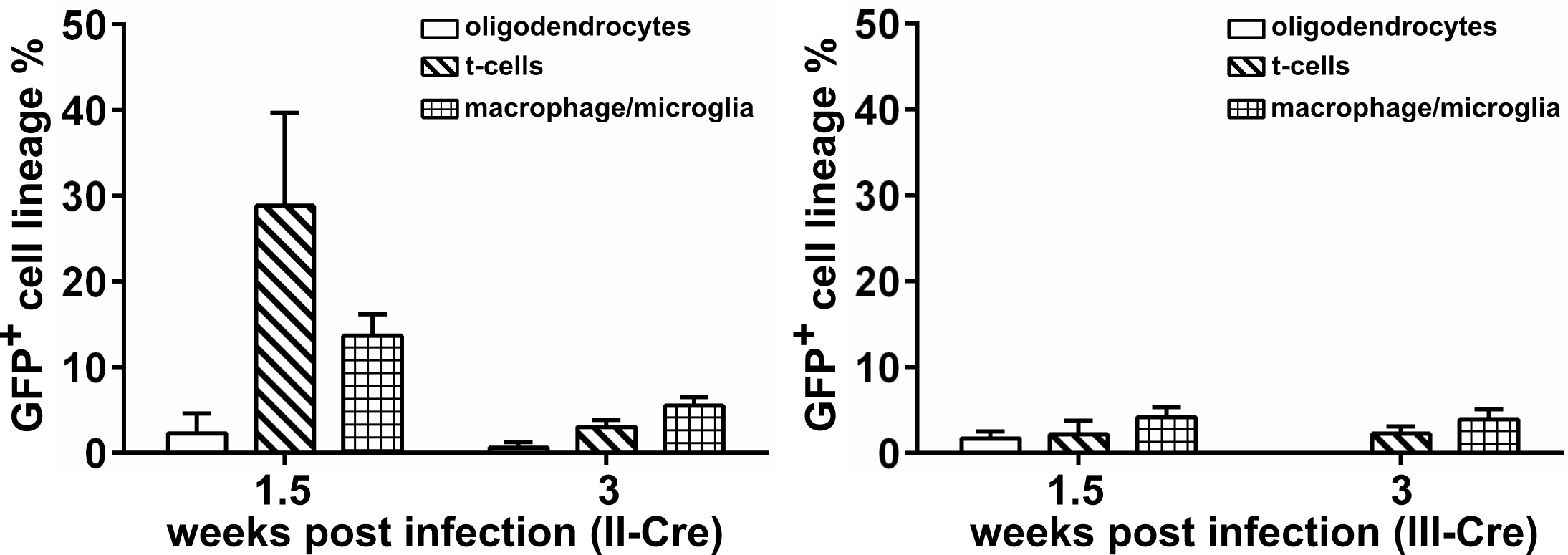

Supplement: S2 Fig — Brain sections from the same mice represented in Fig 1 were stained for oligodendrocytes (anti-Olig2) or T-cells (anti-CD3e) and macrophages/microglia (anti-Iba1). Stained sections were analyzed by confocal microscopy to identify GFP co-localization with described stains. Graphs show the percentage of GFP+ cells identified as oligodendrocytes, T-cells, or macrophages/microglia at specified time points for II-Cre (left graph) or III-Cre (right graph) infected mice. No GFP+ cells co-localized with Olig2 staining at 3 wpi in III-Cre infected mice. Bars, mean ±SEM. In II-Cre-infected mice, for oligodendrocytes, N = 62–111 GFP+ cells examined/ infected mouse, 3 mice/time point (total of 229–317 GFP+ cells evaluated/time point). For T-cells and macrophages/microglia, N = 57–163 GFP+ cells examined/ infected mouse, 3 mice/time point (total of 229–317 GFP+ cells evaluated/ time point.) In III-Cre-infected mice, for oligodendrocytes, N = 100–128 GFP+ cells examined/ infected mouse, 4 mice/time point, (total of 438–447 GFP+ cells evaluated/time point.) For T-cells and macrophage/microglia, N = 100–163 GFP+ cells examined/ infected mouse, 4 mice/time point, (total of 432–520 GFP+ cells per time point.) (TIF) [file ppat.1005447.s002.tif]

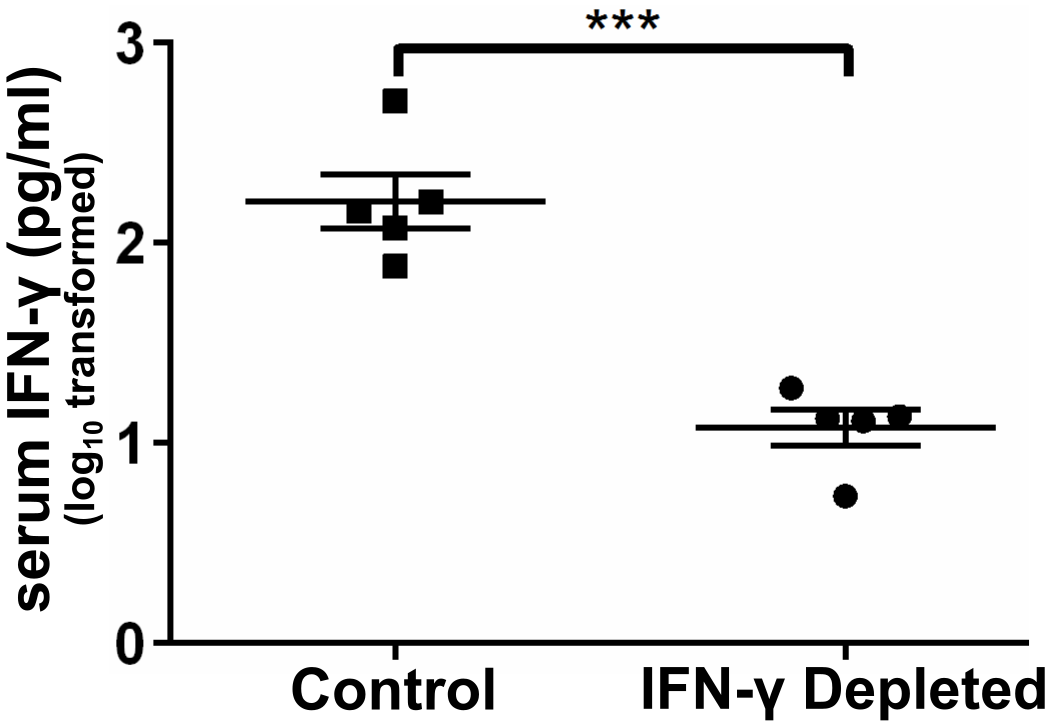

Supplement: S3 Fig — Serum IFN-γ levels determined by specific ELISA. N = 4–5 mice/ treatment. Bars, mean ±SEM. ***p< 0.001 by independent sample, two-tailed t-test. (TIF) [file ppat.1005447.s003.tif]

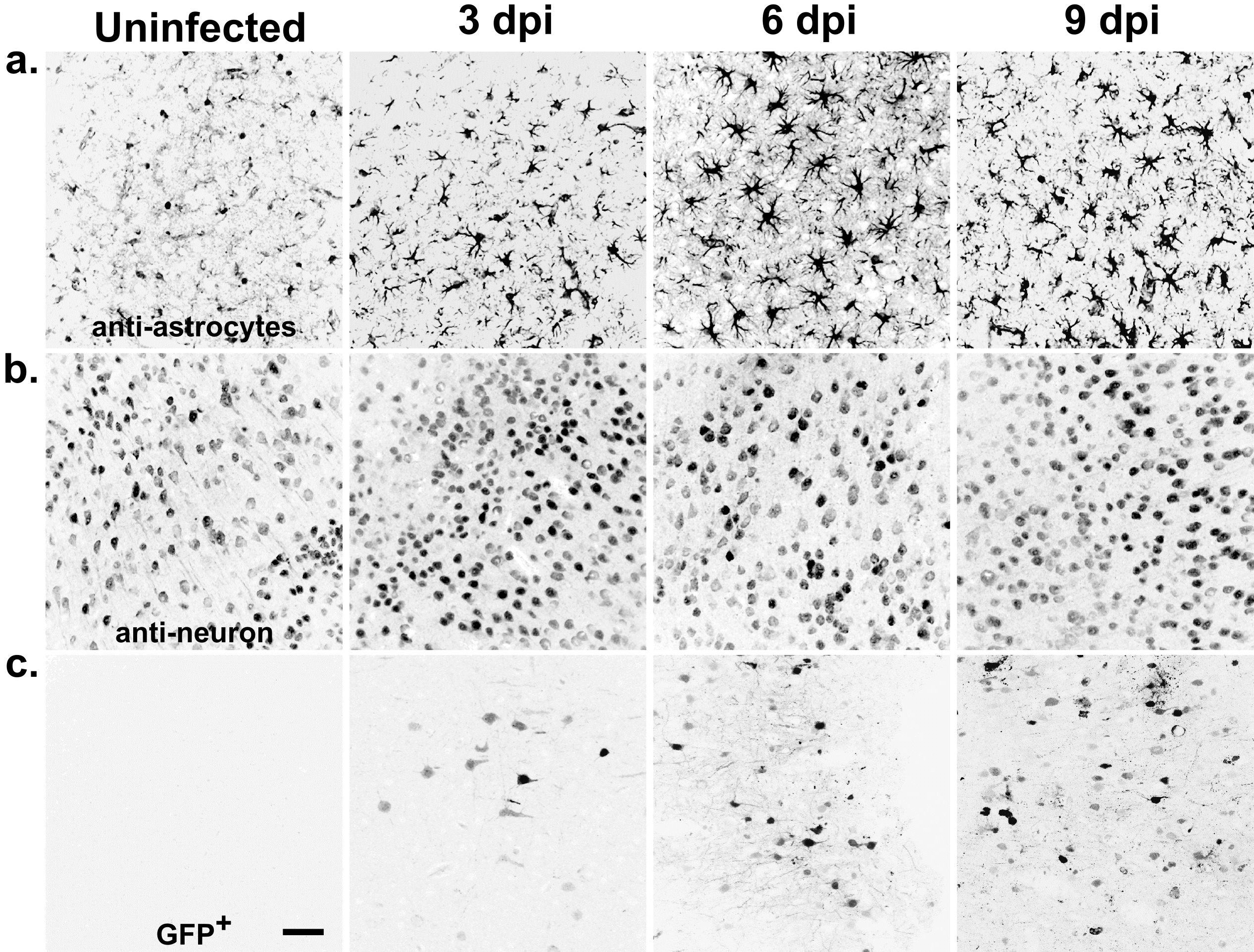

Supplement: S4 Fig — Forty micron brain sections are stained with antibodies against astrocyte proteins (anti-astrocyte) and/or neuronal proteins (anti-neuron). Representative inverted-color, maximal projection images from 8 μm stack of cells stained with (a) anti-neuron or (b) anti-astrocyte stains or (c) with GFP expression at labeled time points. As astrocytes in uninfected mice express little GFAP, brain sections analyzed for these purposes were stained with anti-GFAP, anti-S100β, and anti-ALDL1H1. Anti-S100β stains astrocytic nuclei/cytoplasm, anti-ALDL1H1 stains astrocytic cytoplasm, and anti-GFAP stains astrocytic processes. Note that for astrocytes, progressing from uninfected to 9 dpi, processes that stain with anti-GFAP antibodies are more clearly identified but still do not overlap in space. Scale bar, 50 μm. (TIF) [file ppat.1005447.s004.tif]

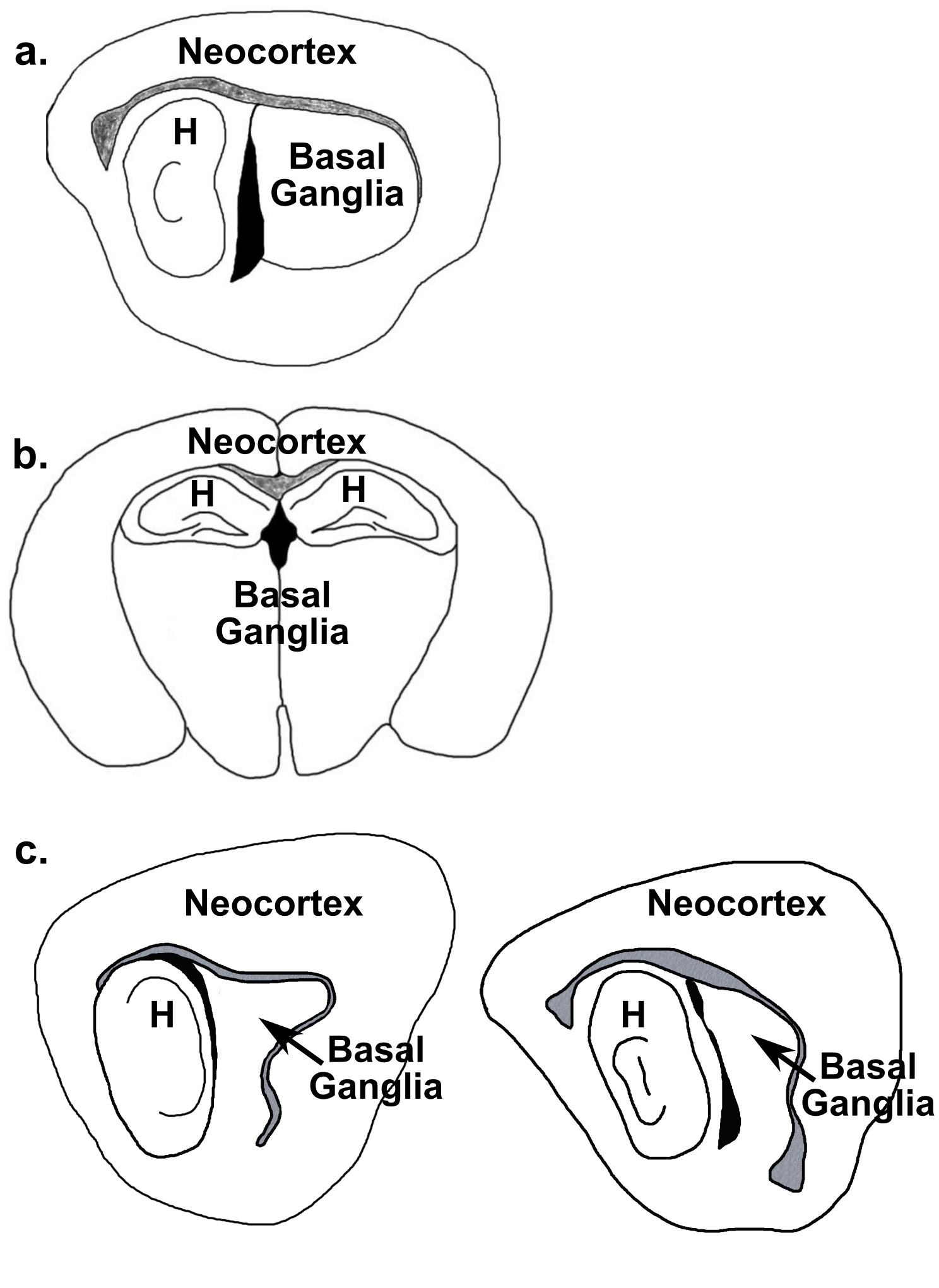

Supplement: S5 Fig — (a) Schematic of sagittal brain section shown in Fig 1a. (b) Schematic of coronal brain section(s) shown in Fig 2a. Whole brain section is drawn here while Fig 2a shows two hemi-sections placed next to each other. (c) Schematics of sagittal brain sections shown in Fig 3a and 3b. Major brain areas are labeled. H = hippocampus. Gray shading represents the corpus collosum, a major white matter tract. Black shading represents ventricular space filled with cerebrospinal fluid. (TIF) [file ppat.1005447.s005.tif]

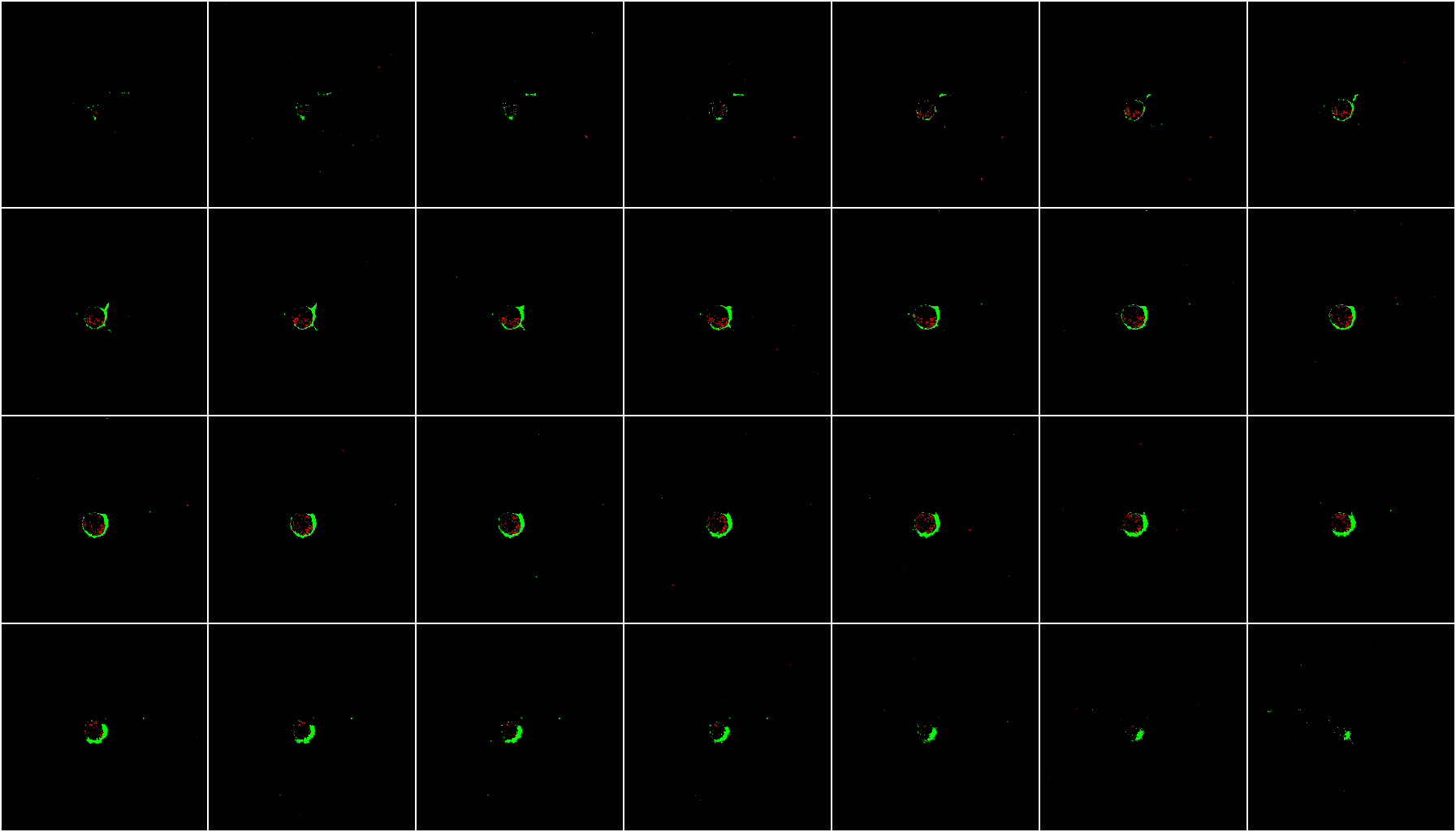

Supplement: S6 Fig — (TIF) [file ppat.1005447.s006.tif]
